# Causal Conditionals, Tendency Causal Claims and Statistical Relevance

**DOI:** 10.1007/s13164-024-00725-0

**Published:** 2024-02-28

**Authors:** Michał Sikorski, Noah van Dongen, Jan Sprenger

**Affiliations:** 1 Center for Philosophy, Science, and Policy, Department of Biomedical Sciences and Public Health, Marche Polytechnic University, Ancona, Italy; 2 University of Amsterdam, Amsterdam, Netherlands; 3 Center for Logic, Language and Cognition (LLC), Department of Philosophy and Education, University of Turin, Turin, Italy

## Abstract

Indicative conditionals and tendency causal claims are closely related (e.g., Frosch and Byrne, 2012), but despite these connections, they are usually studied separately. A unifying framework could consist in their dependence on probabilistic factors such as high conditional probability and statistical relevance (e.g., Adams, 1975; Eells, 1991; Douven, 2008, 2015). This paper presents a comparative empirical study on differences between judgments on tendency causal claims and indicative conditionals, how these judgments are driven by probabilistic factors, and how these factors differ in their predictive power for both causal and conditional claims.

## Introduction and Theoretical Background

Indicative conditionals—that is, conditionals that do not involve the auxiliary verb “would”—are important linguistic structures. Among other things, we use them to predict and explain events, to formulate instructions, and to describe causal relationships. For example, we can describe a *causal tendency*, i.e., the general tendency of a cause to bring about an effect, using explicit causal wording:

- A lot of rain causes the ground to be waterlogged.

but also by means of an indicative conditional (henceforth “conditional”)

1. If it rains a lot, then the ground will be waterlogged.

Similarly, a tendency causal claim^1^ like

- Pressing the red button causes the fire alarm to go off.

can be rephrased using the conditional

(2b) If you press the red button, the fire alarm goes off.

(example taken from Declerck and Reed 2012). In general, indicative conditionals seem to correspond systematically to tendency causal claims (see also Experiment 2 and 3 in Over et al. 2007). Whoever accepts (1a) or (2a) may also be inclined to accept the corresponding indicative conditionals (1b) and (2b), or vice versa.

In the above examples such as (1a/b) and (2a/b), the link between cause and effect was quite strong: given the cause, the effect would occur almost certainly. However, some tendency causal claims express a weaker relationship: the cause *raises the probability of the effect*, but the effect may not be likely even in the presence of the cause. An example of a such case is:

- Smoking causes lung cancer.

Most people would probably classify this sentence as true, but the corresponding conditional

(3b) If one smokes, one will get lung cancer.

seems false, or at least much less plausible than (3a).

So it seems that causal claims and conditionals are not always evaluated in the same way. Specifically, the difference between (3a) and (3b) suggests that true (tendency) causal claims are not always evaluated as true conditionals, where we seem to require that lung cancer will occur with high probability, too.

In this paper, we focus on tendency causal claims such as (1a), (2a) and (3a) and investigate how their evaluation as true (or highly acceptable) differs from the evaluation of the corresponding indicative conditionals. Specifically, we study the role of probability in driving such evaluations: can judgments on the conditional and unconditional probability of the effect predict whether we classify the causal claim and/or the indicative conditional as true or false? And can the correlation between classifications and probability judgments also *explain* the differences between causal claims and conditionals (e.g., in terms of concepts such as statistical relevance and high conditional probability)?

This project is interesting for multiple reasons. First, many experiments have been devoted to conditionals and causal claims separately (e.g., Frosch and Byrne 2012; Sloman and Lagnado 2015; Douven 2016). However, as far as we know, there are no experiments devoted directly to testing the relation between both kinds of expressions. Specifically, we study how the assessment of causal strength affects the use of conditionals for reasoning, explaining and decision-making (for studies on the probabilistic aspects of causal conditionals, see Oberauer and Wilhelm 2003; Over et al. 2007; Over 2017).

Second, both in theories of causality and of indicative conditionals, probability plays an important role. Indeed, on the theoretical level, probability-raising (i.e., statistical relevance) has been identified as a key feature of tendency causal claims (e.g., Suppes 1970; Eells 1991) and in quantifying the strength of a causal connection (e.g., Cheng 1997; Fitelson and Hitchcock 2011; Sprenger 2018). On the other hand, probabilistic accounts of conditionals have analyzed their meaning in terms of the conditional probability of the consequent, given the antecedent (e.g., Ramsey 1926; Adams 1975; Edgington 1986; 1995). We analyze whether or not these theoretical differences are empirically traceable in the evaluation of causal claims and conditionals.

Third, the project may be of interest to psychologists and linguists working on conditionals. A large part of the empirical literature on probability and conditionals has focused on conditionals, which express a reasoning process in the speaker’s mind, for example, “if this paper was rejected, then it must have been bad” (e.g., Johnson-Laird and Byrne 2002). The consequent of these conditionals can typically be preceded by the phrase “then it means that” or the modal auxiliary “must”. A second category are causal conditionals where the antecedent expresses an actual or potential cause of the consequent, expressing a straightforward causal connection between antecedent and consequent, such as “if the students don’t prepare well, the exam will go badly”.^2^ Dancygier (1998, 2003) calls the first group of conditionals “inferential conditionals” while the second group falls under the heading (but is not identical to) “content conditionals”.^3^ The results of our paper contribute to these research programs by investigating the truth and acceptability conditions of causal conditionals.

Fourth, the project sheds, as a byproduct, light on a debate between two theories of the plausibility and acceptability of indicative conditionals: accounts where the acceptability of the conditional “if *C*, then *E*” follows the conditional probability *p*(*E*|*C*) (Adams 1975; Evans et al. 2007; Over et al. 2007; Egré and Cozic 2011; Over 2017), and accounts such as Evidential Support Theory (EST: Douven 2008; 2015; Krzyżanowska 2015; 2017) which demand that for a conditional to be acceptable, (i) *p*(*E*|*C*) be high, and (ii) $p(E|C) > p(E)$. In other words, *C* must also *raise* the probability of *E*. Quantitatively precise versions of EST are given by van Rooij and Schulz (2019) and Crupi and Iacona (2021). In the next section, we explain the hypotheses of our paper in greater detail.

## The Hypotheses

The baseline idea of our paper is that judgments on the truth value or acceptability of a conditional can be predicted on the basis of judgments on the corresponding causal claim and probabilistic factors. While this claim is consistent with most of the theoretical and empirical literature, it is too vague to be tested experimentally. We will therefore split it up into several more precise hypotheses.

Our first hypothesis concerns the logical relationship between conditionals and the corresponding tendency causal claims. Two different kinds of relations are possible:

1. (Necessity) Conditionals are classified as true *only if* the corresponding tendency causal claim is classified as true.
2. (Sufficiency) Conditionals are classified as true *if* the corresponding tendency causal claim is classified as true.

In light of the discussed examples and previous empirical research (e.g., Skovgaard-Olsen et al. 2016a; b; Skovgaard-Olsen et al. 2017; Douven et al. 2018; Skovgaard-Olsen et al. 2019), we expect that H1.a will be supported. We contrast it with the less plausible H1.b. In the light of examples such as (3a/b), we would expect that H1.b fails in empirical investigation. In many cases, the truth of both expressions will co-occur but some conditionals are expected to be classified as false although the corresponding tendency causal claim appears true (e.g., “smoking causes cancer”). High conditional probability of the consequent given the antecedent is plausibly one of the conditions of acceptability of conditionals (Over et al. 2007; Douven and Verbrugge 2012), but it is not required for classifying a tendency causal claim as true, as we have seen in the (3a/b) example. This speaks against H1.b.

We operationalize these hypotheses by demanding that of all conditionals evaluated as true, only a small percentage of the corresponding tendency causal claims are evaluated as false (H1.a). Similarly, for an overwhelming percentage of all tendency causal claims evaluated as true, the same claim in conditional form needs to be evaluated as true (H1.b). For the respective thresholds we consider a *strict interpretation* (5 and 95%) and a *lenient interpretation* (10 and 90%).^4^

The next hypothesis regards the question of whether *p*(*E*|*C*) is a strong predictor of the plausibility that a conditional is true (Handley et al. 2006; Evans et al. 2007; Over et al. 2007; Over 2017; Over and Cruz 2023; contrary evidence is presented in Douven and Verbrugge 2010; Skovgaard-Olsen et al. 2016b). However, even critics concede a *weak version* of Adams’s Thesis, i.e., *p*(*E*|*C*) is highly correlated with the classification of a conditional as true (e.g., Douven and Verbrugge 2010, p. 306). We should therefore expect that this probability predicts the classification of a conditional at least to some degree.

H2.a (Weak Adams’s Thesis) The conditional probability *p*(*E*|*C*) predicts the classification of conditionals of the form “if *C*, then *E*” as true or false, and its degree of acceptability.

Suppose, however, that H2.a is *not* confirmed, or only weakly so. It could then be of interest to investigate whether it holds *when restricted to causal claims classified as true*. Indeed, the experiments by Skovgaard-Olsen et al. (2016b) show strong correlations between judgments on conditionals and *p*(*E*|*C*) *only when there is a clear relevance between cause and effect*. H2.b operationalizes a “relevantist” account of conditionals along these lines, i.e., they are classified as true if and only if (1) the corresponding causal claim is classified as true; and (2) *p*(*E*|*C*) is “high enough”.

H2.b (Restricted Adams’s Thesis) In the class of tendency causal claims classified as true/highly acceptable, the conditional probability *p*(*E*|*C*) predicts the evaluation of the conditional “If *C*, then *E*” as true/highly acceptable.

As a criterion for evaluating H2.a and H2.b, we adopt the statistical significance of including conditional probability as a predictor variable, plus a non-negligible effect size. Effect size is measured by how much variance in the data can be explained by the predictor variables and expressed numerically by the squared correlation coefficient $R^2$. For an effect size to be meaningful, we demand that it exceed the value $R^2 = 0.09$, which is conventionally identified with the lower bound of a medium effect (Cohen 1988).

The remaining hypotheses concern the role of statistical relevance in the evaluation of tendency causal claims and conditionals, as predicted by probabilistic accounts of causal strength, Evidential Support Theory and the various covariation proposals.

In the context of conditionals and causal claims, statistical relevance is typically measured by a function of two arguments, increasing in the first argument ($x = p(E|C)$) and decreasing in the second argument ($y = p(E|\lnot C)$ or $y = p(E)$). The most common ways of combining these arguments are as follows:

$$\begin{aligned} d(x, y)&= x-y&\quad r(x, y)&= \log (x/y) \\ z(x, y)&= \frac{x-y}{1-y}&\quad l(x, y)&= \log \frac{x}{1-x} - \log \frac{y}{1-y} \end{aligned}$$

which are known, respectively, as the difference measure *d*, the log-ratio measure *r*, the *z*-measure or normalized difference measure, and the log-likelihood measure *l*. Dependent on whether the second argument is $y = p(E)$ or $y = p(E|\lnot C)$, the difference measure *d* reads either $d = p(E|C) - p(E)$, or $d = p(E|C) - p(E|\lnot C)$, and both measures have been defended as quantification of causal strength (Suppes 1970; Pearl 2001; Sprenger 2018; Sprenger and Hartmann 2019). Crupi and Iacona (2021) propose *z* (with $y = p(E)$) as a measure of the acceptability of an indicative conditional, and so do van Rooij and Schulz (2019) (but with $y = p(E|\lnot C)$). The peculiar feature of the *z*-measure is that the probability raise is set in relation to the maximal possible raise, and so the degree of statistical relevance is always *at least as high as the conditional probability* $x = p(E|C)$. In other words, the *z*-measure captures aspects of high conditional probability and statistical relevance in a single number.^5^

On the basis of these measures, we can examine a series of hypotheses about how statistical relevance affects judgments on the truth or acceptability of causal and conditional claims:

H3.a Statistical relevance measures predict the classification of tendency causal claims as true or false (respectively their degree of acceptability).
H3.b Statistical relevance measures predict the classification of a conditional as true or false (respectively their degree of acceptability).
H3.c In the class of tendency causal claims classified as true/highly acceptable, statistical relevance measures predict the evaluation of conditional claims as true/highly acceptable.
H3.d Statistical relevance and conditional probability are, taken together, better predictors for the classification of a conditional than conditional probability alone.

We expect the first hypothesis, H3.a, to come out confirmed since the increase in probability (upon intervention of the cause) has a strong theoretical basis as a predictor of causal strength, as explained above. H3.b tests probabilistic accounts of conditionals centered on evidential support and statistical relevance, such as van Rooij and Schulz (2019) and Crupi and Iacona (2021). H3.c tests the same hypothesis, restricted to the class of tendency causal claims classified as true (i.e., when we know that the cause is relevant for the effect). Finally, H3.d tests whether taking into account statistical relevance on top of conditional probability improves the prediction of the classification of the conditional.

If one of H3.b/c/d came out confirmed, it would give a boost to Evidential Support Theory and similar accounts that stress the importance of statistical relevance. If not, it might not affect their normative significance but diminish their predictive value.

The hypotheses H3.a-H3.c are evaluated on the same basis as before: adding statistical relevance as a predictor variable needs to be statistically significant, and the effect size as measured by the correlation coefficient must exceed $R^2 = 0.09$. For H3.d, we ask an increase in explained variance by $9\%$ i.e., and increase in $R^2$ of 0.09 over the results of hypothesis H2.a.

## Experiment 1

### Participants

Participants were recruited via Amazon’s Mechanical Turk (www.mturk.com). Mechanical Turk directed the participants to the experiment that was run on the Qualtrics platform (www.qualtrics.com). In return for their participation, subjects received a small monetary compensation. Seventy-four native English speakers participated in the experiment. Eighteen participants were excluded because they failed to give the correct response to at least one of the control questions. All participants indicated to have participated seriously. At the end of the experiment, participants were asked an open question about what they thought the experiment was about. None of the participants displayed clear knowledge of the purpose of the experiment. In total, 56 [39 female, mean age = 40.59 years, s.d. = 11.69 years] participants were included in the analysis.

### Design

We used a within-subjects design were each participant evaluated 19 vignettes. These vignettes were presented in random order on the participants’ computer screen. The participants were instructed to answer questions with the requirement that each question needed to be answered to be able to progress to the next item (i.e., forced-choice). The entire experiment was conducted in English.

Control questions were used as a check on participants’ attention and participation. Randomly dispersed throughout the experiment, participants had to give a correct answer to several repeats of the *elimination questions* “For quality control, please select answer category five. If you do not select five, the survey will be terminated.” In addition, subjects had to rate on a five-point Likert scale how seriously they participated in the experiment (1= “completely unserious”, 5 = “completely serious”). We included an open question on the purpose of the experiment because knowledge of the experiment could influence the behaviour of participants. Our exclusion criteria were: failure to give the answer 5 on one of the elimination questions; rating their seriousness in participation as 3 or lower; or describing the experiment as being about conditionals and causation or something similar.

### Material and Procedure

Participants had to evaluate tendency causal claims and the corresponding conditional claims in hypothetical vignettes, where a certain situation was described (e.g., the effect of prohibiting alcohol on the crime rate). We used content conditionals which can be interpreted causally. Such conditionals are usually called *causal conditionals*—not in the strong, ontological sense that there is actually a causal connection between antecedent and consequent, but rather in the weaker, epistemic sense that such conditionals “can be justified by evidence about a possible causal relation or mechanism” (e.g., Over et al. 2007, p. 65). Experimental studies using such conditionals are both accepted and common in the literature (see e.g., Oberauer and Wilhelm 2003; Over et al. 2007; Over 2017).^6^

Through several pre-studies, 19 vignettes were selected on comprehensibility from a list of 60. When directed from Mechanical Turk to Qualtrics, participants first received instructions on the experiment. After the instructions they were presented with the vignettes in a randomized sequence. The 19 vignettes consisted of four questions each, eliciting probability judgments as well as dichotomous judgments on causal and conditional claims: **(Unconditional) Probability of Consequent** This question elicits the probability of a certain development without making specific assumptions (e.g., “how likely is it that the crime rate will decline in the next five years?”). **Conditional Probability of the Consequent** This question elicits the probability of the same development under a specific assumption stated in the antecedent (e.g., “how likely is it that the crime rate will decline in the next five years if alcohol consumption is made illegal?”).^7^**Causal Claim** This question asks the participants to evaluate the truth or falsity of the causal connection between antecedent and consequent (e.g., “Making alcohol consumption illegal will cause the crime rate to decline in the next five years”). **Conditional Claim** This question asks the participants to evaluate the truth or falsity of the corresponding indicative conditional (e.g. “If alcohol consumption is made illegal, then the crime rate will decline in the next five years.”)

The first two questions had to be answered on a visual analog scale of probability percentages from 0% to 100%. The third and fourth question had to be answered with either ”true” or ”false”. We reproduce the experimental material in Appendix A and B. This formulation of the stimuli implies that we will calculate statistical relevance measures with $x = p(E|C)$ and $y = p(E)$; see Experiment 2 for an extension to $y = p(E|\lnot C)$.

### Results

#### Hypotheses H1.a and H1.b: Conditional vs. Causal Claims

Combined, the data consisted of 1064 entries; 56 participants responded to 19 vignettes. We evaluated H1.a and H1.b by simply checking the frequency statistics for the relevant categories (see Table 1). Of the 531 data points where a conditional was classified as true, 25 data points classified the corresponding tendency causal claim as false. This corresponds to a percentage of 4,71% and therefore confirms our hypothesis H1.a that perceived presence of a causal relationship is *necessary* for classifying a conditional as true, both for the strict 5% and the lenient 10% threshold. By contrast, Hypothesis H1.b that classifying a causal claim as true is a *sufficient* condition for classifying the conditional as true was not borne out by the data: of 611 data points where the tendency causal claim was evaluated as true, only 506 evaluated the corresponding conditional as true. This percentage of 82,82% is clearly below the thresholds of 90% and 95% necessary to establish sufficiency.

**Table 1:** Classification of causal and conditional claims as true and false

Contingency Table		Conditional Claim		Total
		True	False	
Causal Claim	True	506 [82.82%]	105 [17.18%]	611 [100.00%]
		[95.29%]	[19.70%]	
	False	25 [5.52%]	428 [94.48%]	453 [100.00%]
		[4.71%]	[80.30%]	
	Total	531	533	1064
		[100%]	[100%]

**Table 2:** The Generalized Linear Mixed Model (GLMM) for the dependent variable *Causal Claim* as a function of *Conditional Probability*

Type of Effect	Variable	Coefficient	Std. Error	z	p
Fixed effects	intercept	-4.61	0.46	-10.13	< 0.0001
	Conditional Probability	0.076	0.006	13.06	< 0.0001
Random effects	Participant	1.84	1.36		
	Vignette	0.64	0.80		
Explained variance: $R^2 = 0.34$. Residual degrees of freedom = 1060.					

#### Hypothesis H2.a and H2.b: Weak and Restricted Adams’s Thesis

To test H2.a and H2.b, a Generalized Linear Mixed Model (GLMM) was used. We used a logit link function, as the outcome variable for each hypothesis was binary (0 = False, 1 = True). We added participants and vignette number as crossed random effects, because of difference in content between the vignettes, and possible differences in their interpretation between participants. We used the R package *lme4* (Bates et al. 2015) to estimate the GLMM’s regression coefficients, variance components, and the amount of variance in the outcome explained by the predictors (i.e., *marginal* $R^2_{GLMM}$, $R^2$ onwards; Nakagawa and Schielzeth 2013).

For hypothesis H2.a, the results show a strong and positive association between the conditional probability attributed to the consequent of the conditional, and the log-odds of the corresponding conditional being considered as true. Specifically, with every percentage-point increase in conditional probability these log-odds are estimated to increase by 0.07 (an increase of 7 over the full 100 percentage points). Most importantly, the model explains 48% ($R^2=0.48$) of the variance in the participants tendency to indicate conditionals as either true or false (see Table 2). In short, H2.a is supported by the observed data.

To test hypothesis H2.b, only those data points were used where the tendency causal claim was indicated as true (see Table 1). Similar to the results for H2.a, results show a positive association between the conditional probability attributed to the consequent of the conditional, and the log-odds of corresponding conditional being considered as true (see Table 3). Although the amount of variance explained is greatly reduced, from 48% to 12%, it is still considered meaningful ($R^2>0.09$) and thus supporting H2.b.

**Table 3:** The Generalized Linear Mixed Model (GLMM) for the dependent variable *Conditional Claim* as a function of *Conditional Probability* when *Causal Claim = “true”*

Type of Effect	Variable	Coefficient	Std. Error	z	p
Fixed effects	intercept	0.89	0.62	-1.14	0.15
	Conditional Probability	0.05	0.008	5.79	< 0.0001
Random effects	Participant	3.39	1.84		
	Vignette	0.45	0.67		
Explained variance: $R^2 = 0.13$. Residual degrees of freedom = 607.					

#### Hypotheses H3.a—H3.d: The Impact of Statistical Relevance

As above, a Generalized Linear Mixed Model (GLMM) was used to test hypothesis H3.a, H3.b, H3.c, and H3.d. For H3.a, all statistical relevance measures except *z* predict the classification of tendency causal claims as true or false (see Table 4). For the statistical relevance measures *d*, *r*, and *l*, the analyses show a positive and meaningful association ($R^2 > 0.09$) with the proclivity of participants to assess the tendency causal claim as true. Specifically, the coefficients indicate the estimated increase in log-odds (per unit of the relevance measure) of tendency causal claims being indicated as true versus false.^8^ The random-effects for vignette and participant, though non-zero, appear to be minor. Specifically, their coefficients are only slightly larger than their standard errors. Based on the test statistics (z-values) and amount of explained variance ($R^2$), the association between *d* and classification of tendency causal claims was the strongest (i.e., largest coefficient with respect to its standard error). The weakest association was with the *z* measure.

**Table 4:** The Generalized Linear Mixed Model (GLMM) for the dependent variable *Causal Claim* as a function of *Statistical Relevance*: the *d*-measure, *r*-measure, *l*-measure, and *z*-measure

Type of Effect	Variable	Coefficient	Std. Error	z	p
Fixed effects	intercept	-0.15	0.31	-0.48	0.63
	*d*	5.85	0.53	11.04	< 0.0001
Random effects	Participant	1.14	1.07		
	Vignette	1.26	1.12		
Explained variance: $R^2 = 0.34$. Residual degrees of freedom = 1060.					
Fixed effects	intercept	0.26	0.35	0.75	0.46
	*r*	1.16	0.14	8.36	< 0.0001
Random effects	Participant	1.02	1.01		
	Vignette	1.76	1.33		
Explained variance: $R^2 = 0.22$. Residual degrees of freedom = 1060.					
Fixed effects	intercept	-0.04	0.32	-0.12	0.90
	*l*	0.84	0.08	10.08	< 0.0001
Random effects	Participant	1.10	1.05		
	Vignette	1.33	1.15		
Explained variance: $R^2 = 0.34$. Residual degrees of freedom = 1060.					
Fixed effects	intercept	-0.24	0.29	-0.84	0.40
	*z*	0.01	0.02	0.55	0.58
Random effects	Participant	0.75	0.87		
	Vignette	2.39	1.55		
Explained variance: $$R^2 < 0.001 $$. Residual degrees of freedom = 1060.					

For hypothesis H3.b, all statistical relevance measures predict the classification of conditionals as true or false (see Table 5). For all the statistical relevance measures, the analyses show a positive and meaningful association ($R^2 > 0.09$) with the tendency of participants to assess the conditional as true, thus supporting hypothesis H3.b.

**Table 5:** The Generalized Linear Mixed Model (GLMM) for the dependent variable *Conditional* as a function of *Statistical Relevance*: the *d*-measure, *r*-measure, *l*-measure, and *z*-

Type of Effect	Variable	Coefficient	Std. Error	*z*	*p*
Fixed effects	intercept	-0.69	0.31	-2.19	0.029
	*d*	5.27	0.48	10.95	< 0.0001
Random effects	Participant	1.51	1.23		
	Vignette	1.18	1.09		
Explained variance: $R^2 = 0.28$. Residual degrees of freedom = 1060.					
Fixed effects	intercept	-0.26	0.33	-0.79	0.43
	*r*	1.02	0.12	8.27	< 0.0001
Random effects	Participant	1.17	1.08		
	Vignette	1.51	1.23		
Explained variance: $R^2 = 0.18$. Residual degrees of freedom = 1060.					
Fixed effects	intercept	-0.61	0.001	-423.6	< 0.0001
	*l*	0.80	0.002	553.8	< 0.0001
Random effects	Participant	1.45	1.20		
	Vignette	1.16	1.08		
Explained variance: $R^2 = 0.31$. Residual degrees of freedom = 1060.					
Fixed effects	intercept	-0.024	0.34	-0.07	0.95
	*z*	0.22	0.08	2.63	0.009
Random effects	Participant	0.94	0.97		
	Vignette	1.79	1.34		
Explained variance: $$R^2 = 0.20 $$. Residual degrees of freedom = 1060.					

To test hypotheses H3.c, only those data points were used where the tendency causal claim was indicated as true (see Table 1). The results show that none of the statistical relevance measures made a meaningful difference in explaining the variance in the participants’ tendency to indicate the conditionals as true or false ($R^2<0.09$ in all cases, see Table 6), thus supporting our conjecture of a null effect and contradicting hypothesis H3.c.

**Table 6:** The Generalized Linear Mixed Model (GLMM) for the dependent variable *Conditional Claim* as a function of *Statistical Relevance* (*d*-measure, *r*-measure, *l*-measure, and *z*-measure) when *Causal Claim = “true”*

Type of Effect	Variable	Coefficient	Std. Error	z	p
Fixed effects	intercept	1.89	0.38	4.99	< 0.0001
	*d*	2.04	0.62	3.28	0.001
Random effects	Participant	2.80	1.67		
	Vignette	0.64	0.80		
Explained variance: $R^2 = 0.04$. Residual degrees of freedom = 607.					
Fixed effects	intercept	2.15	0.37	5.82	< 0.0001
	*r*	0.36	0.16	2.17	0.030
Random effects	Participant	2.72	1.65		
	Vignette	0.65	0.81		
Explained variance: $R^2 = 0.02$. Residual degrees of freedom = 607.					
Fixed effects	intercept	1.89	0.38	4.99	< 0.0001
	*l*	0.37	0.09	4.00	0.0001
Random effects	Participant	2.94	1.71		
	Vignette	0.64	0.80		
Explained variance: $R^2 = 0.07$. Residual degrees of freedom = 607.					
Fixed effects	intercept	1.63	0.38	4.31	< 0.0001
	*z*	0.09	0.05	1.82	0.07
Random effects	Participant	2.99	1.73		
	Vignette	0.56	0.75		
Explained variance: $R^2 = 0.04$. Residual degrees of freedom = 607.					

To test hypothesis H3.d, the change in $R^2$ is assessed when *conditional probability* *p*(*E*|*C*) is added to the models of H3.a. For all statistical relevance measures, none made a meaningful contribution over and above the conditional probability on the prediction of participants’ proclivity to indicate the conditional as true (see Table 7). Specifically, the $R^2$ of these models does not show a large enough increase (at most 0.01) over the models that only include the conditional probability as a prediction (see hypothesis H2.a; Table 2)

**Table 7:** The Generalized Linear Mixed Model (GLMM) for the dependent variable *Conditional Claim* as a function of *Statistical Relevance* (*d*-measure, *r*-measure, *l*-measure, and *z*-measure) and *conditional probability*

Type of Effect	Variable	Coefficient	Std. Error	z	p
Fixed effects	intercept	-4.30	0.47	-9.18	< 0.0001
	*d*	1.32	0.57	2.31	0.02
	Conditional Probability	6.81	0.67	10.23	< 0.0001
Random effects	Participant	1.89	1.38		
	Vignette	0.60	0.77		
Explained variance: $R^2 = 0.48$. Residual degrees of freedom = 1059.					
Fixed effects	intercept	-4.48	0.46	-9.70	< 0.0001
	*r*	0.2760	0.15	1.89	0.06
	Conditional Probability	7.26	0.61	11.87	< 0.0001
Random effects	Participant	1.87	1.37		
	Vignette	0.62	0.79		
Explained variance: $R^2 = 0.49$. Residual degrees of freedom = 1059.					
Fixed effects	intercept	-4.27	0.47	-9.07	< 0.0001
	*l*	0.21	0.09	2.29	0.02
	Conditional Probability	6.80	0.67	10.18	< 0.0001
Random effects	Participant	1.87	1.37		
	Vignette	0.60	0.77		
Explained variance: $R^2 = 0.49$. Residual degrees of freedom = 1059.					
Fixed effects	intercept	-4.60	0.46	-10.03	< 0.0001
	*z*	0.01	0.028	0.41	0.69
	Conditional Probability	7.61	0.59	12.91	< 0.0001
Random effects	Participant	1.85	1.36		
	Vignette	0.64	0.80		
Explained variance: $R^2 = 0.48$. Residual degrees of freedom = 1059.					

### Summary of Results of Experiment 1

The experiment has confirmed that the truth conditions of indicative conditionals are more demanding than the truth conditions of the corresponding tendency causal claims (support for H1.a, no support for H1.b). It has also confirmed the weak, qualitative version of Adams’s Thesis—the conditional probability of the consequent predicts the judgment on the truth value of the conditional (H2.a)—, as well as its restriction to tendency causal claims evaluated as true (H2.b). The hypotheses about statistical relevance enjoy mixed support: statistical relevance predicts the classifications of causal/conditional claims as true (H3.a and H3.b), but this may simply be due to the fact that high statistical general relevance co-varies with high conditional probability *p*(*E*|*C*). Indeed, once we control for this effect, statistical relevance adds no further predictive value (null results for H3.c and H3.d).

## Experiment 2

The second experiment consisted in a conceptual replication of the first experiment, using only continuous scales for the dependent variables, and replacing one of the predictor variables. This experiment consisted of four separate parts (2.A, 2.B, 2.C, and 2.D) with independent samples for each part. Slight alterations were made in the phrasing of the vignettes. That said, 2.A was essentially the same experiment as Experiment 1 whereas in 2.B, 2.C and 2.D, important elements of the response variables were changed. These changes were motivated by two questions that we (and some reviewers) had outlined as targets for future research: First, whether our results would carry over from judgments about the *truth value* of a conditional to its *acceptability* (reasons for suspecting invariance are given byDouven and Krzyżanowska 2018). In other words, we replaced a dichotomous choice (“true/false”) for the evaluation of the causal claim and the conditional by a continuous scale. Second, given that various important statistical relevance measures such as $\Delta {}p = p(E|C) - p(E|\lnot C)$ are calculated on the basis of the probability of the effect given the *negation* of the cause (i.e., $p(E|\lnot C)$), we wanted to see whether such measures would predict judgments on causal claims and conditionals any better (or worse) than measures which depend on $x = p(E|C)$ and $y = p(E)$.

### Participants

Similar to the first experiment, participants were recruited via Amazon’s Mechanical Turk (www.mturk.com). Mechanical Turk directed the participants to the experiment that was run on the Qualtrics platform (www.qualtrics.com). In return for their participation, subjects received a small monetary compensation. 77 people participated in Experiment 2.a, 75 participated in Experiment 2.b, 75 participated in Experiment 2.c, and 72 participated in Experiment 2.d. In total 38 participants were excluded (6 from 2.a, 12 from 2.b, 5 from 2.c, 15 from and 2.d), because they failed to give the correct response to at least one of the control questions. All participants indicated to have participated seriously. None of the participants displayed clear knowledge of the purpose of the experiment on the open question about what they thought the experiment was about. Thus, the number of participants included in the analysis was 71 for Experiment 2.a, 63 for Experiment 2.b, 70 for Experiment 2.c, an 57 for Experiment 2.d.

### Design

We used the same within-subject design were each participant evaluated 18 vignettes. These vignettes were presented in random order on the participants’ computer screen and they were instructed to answer questions with the requirement that each question needed to be answered to be able to progress to the next item (i.e., forced-choice)

### Material and Procedure

Similar to Experiment 1, this experiment consisted of hypothetical vignettes, which the participants had to evaluate. With respect to Experiment 1, we altered the phrasing of the four questions in each vignette. One vignette had to be dropped, because it could not be fitted to the alterations. For a complete list of the altered vignettes, see Appendix D. The difference between the experiments is represented schematically in Table 8.

**Table 8:** Differences between Experiments 2.A, 2.B, 2.C and 2.D in terms of the quantities they elicit

Experiment	Classification of causal and conditional claims	*p*(*E*|*C*)?	*p*(*E*)?	$p(E|\lnot C)$?
2.A (=1)	dichotomous scale (true/false)	yes	yes	no
2.B	dichotomous scale (true/false)	yes	no	yes
2.C	continuous scale (0-100 agreement)	yes	yes	no
2.D	continuous scale (0-100 agreement)	yes	no	yes

Across all parts of this experiment (2.A—2.D) the B question (“Conditional Probability of the Consequent” in Experiment 1) was rephrased to eliminate potential ambiguities with respect to the probability of the conditional (compare the discussion in footnote 3 on page 9). Specifically, this question was rephrased to have structure: “Suppose *x*. How likely is it that *y*?”. For example, “Suppose that alcohol consumption will be prohibited. How likely is it then that the crime rate will decline in the next 5 years?”

In Experiment 2.B and 2.D, the A question of Experiment 1 was replaced in order to elicit $p(E|\lnot C)$. Instead of a question about unconditional probability of the vignette, an opposite to the B question was presented to the participants. For instance, “Suppose that alcohol consumption will stay legal. How likely is it then that the crime rate will decline in the next 5 years?”

In Experiment 2.C and 2.D, answer options were changed in order to measure the classification of causal claims and conditionals on a continuous scale. In these experiments, the C (causal claim) and D (conditional claim) questions had to be answered on a visual analog scale from 0 to 100. Specifically, participants were asked the extent of their agreement with the claims. For instance:

“Making alcohol consumption illegal causes the crime rate to decline over the next 5 years.”To what extent do you agree with this statement? (0 = completely disagree; 100 = completely agree)

### Results

#### Hypotheses H1.a and H1.b: Conditional vs. Causal Claims

For this hypothesis, only Experiment 2.A and 2.B could be evaluated. In Experiment 2.C and 2.D, causal and conditional claims were no longer answered as ‘true’ or ‘false’, which precluded them from testing this hypothesis. For Experiment 2.A, 11.48% (94 out 819) of true conditional claims have a corresponding false causal claim and 86.21% (725 out of 841) of true causal claims have a corresponding true conditional claim. These results do not support hypotheses H1.a and H1.b. For Experiment 2.B, 9.12% (77 out of 844) of true conditional claims have a corresponding false causal claim and 87.06% (767 out of 881) of true causal claims have a corresponding true conditional claim (see Table 9). These results support hypothesis H1.a at the 10% level, but do not support hypothesis H1.b. Our findings are thus in general agreement with Experiment 1.

**Table 9:** Classification of causal and conditional claims as true and false

		Experiment 2.A			Experiment 2.B		
Contingency Table		Conditional Claim		Total	Conditional Claim		Total
		True	False		True	False	
Causal Claim	True	725	116	841	767	114	881
	False	94	361	455	77	194	271
Total		819	477	1296	844	308	1152

#### Hypotheses H2.a and H2.b: Weak and Restricted Adams’s Thesis

Similar to Experiment 1, a Generalized Linear Mixed Model^9^ was used to test hypotheses H2.a and H2.b. In the case of hypothesis H2.a, the conditional probability explained a significant percentage of variance in the participants responses to the conditional claim across all experiments (2.A:$R^2=0.19$, 2.B:$R^2=0.20$, 2.C:$R^2=0.56$, 2.D:$R^2=0.62$, and all p-values < 0.05). In short, H2.a is supported by the observed data.

To test hypotheses H2.b, only those data points were used where the tendency causal claim was indicated as true (Experiment 2.A and 2.B) or got an agreement score above 80 our of 100 (Experiment 2.C and 2.D). This threshold has been chosen because according to the so-called Lockean Thesis (e.g., Foley 2009) one *accepts* a proposition when it is highly probable, and 80% seems to us a natural (though in no way special) implementation of that criterion. Results show a positive association between the conditional probability attributed to the consequent of the conditional, and the log-odds of corresponding causal conditional being considered as true. However, the amount of variance explained is greatly reduced, and only two of the four experiments show an $R^2 > 0.09$ (2.A:$R^2=0.05$, 2.B:$R^2=0.02$, 2.C:$R^2=0.16$, 2.D:$R^2=0.14$, and all $p < .05$). Thus H2.b is only partially supported.

#### Hypotheses H3.a—H3.d: The Impact of Statistical Relevance

To test H3.a/b/c/d, a GLMM was used analogous to Experiment 1. For hypothesis H3.a, all statistical relevance measures predict the participants response to the tendency causal claims (true or false for Experiments 2.A and 2.B, and level of agreement in Experiments 2.C and 2.D) to a certain extent. However, the amount of variance explained by the statistical relevance measures varies across the experiments.

Hypothesis H3.a was generally not supported, because only in two out of 16 instances did the four statistical relevance measures meet the inference criteria across the four experiments. These cases were *r* ($R^2=0.12$, $p<0.0001$) and *l* ($R^2=0.13$, $p<0.0001$) in experiment 2.D. In general, the statistical relevance measures met the statistical significance criterion ($p < .05$; as it can be expected in a large sample), but failed the effect size criterion $R^2>0.09$. When the effect size criterion was not met, $R^2$ ranged from 0.0002 to 0.08. The *z* measure in particular fared poorly, contrary to theoretical expectations (van Rooij and Schulz 2019; Crupi and Iacona 2021). It delivered statistically significant results only for experiment 2.B and 2.D and had the lowest $R^2$ values of all measures (0.0002, 0.05, 0.002, and 0.004 respectively).

We made a similar finding for hypothesis H3.b: it was supported in two out of sixteen instances (four statistical relevance measures across four experiments).

To test hypothesis H3.C, only those data points were used where the tendency causal claim was indicated as true (Experiment 2.A and 2.B) or got an agreement score above 80 our of 100 (Experiment 2.C and 2.D). In this case, none of the statistical relevance measures adequately predicted the participants response to the conditionals (true or false for Experiments 2.A and 2.B, and level of agreement in Experiments 2.C and 2.D). None of the relevance measures met the $R^2>0.09$ criterion and the $p<0.05$ criterion was only met by *d*, *r*, and *l* in experiment 2.A and *l* in experiment 2.C.

To test hypothesis H3.d, the change in $R^2$ is assessed when *conditional probability* *p*(*E*|*C*) is added to the models of H3.a. Unfortunately, none of the statistical relevance measures is an adequate proxy for the probability effects. For experiment 2.C and 2.D, including a statistical relevance measure even a *negative* effect on the participants’ level of agreement with the conditional.

### Summary of Results of Experiment 2

The results agree with the findings of Experiment 1 regarding H1.a and H1.b: conditional claims have more demanding truth conditions than causal claims. Also, like in the previous experiment, Weak Adams’s Thesis (H2.a) was supported, and its restriction to causal claims (H2.b) enjoys partial support. Regarding the predictive value of statistical relevance, the findings are even more negative than in Experiment 1: none of the four hypotheses has been confirmed, for H3.b–H3.d no effect is visible, and there is just very partial confirmation of H3.a (statistical relevance predicts the classification of the causal claim). This confirms that statistical relevance by itself should be treated with great caution as a predictor of causal and conditional judgments.

## General Discussion

There is a natural mapping between tendency causal claims (“Smoking weed causes dizziness”) and conditionals in the indicative mood (“if somebody smokes weed, she will feel dizzy”). In the presented study, we tested various hypotheses about the classification of such sentences as true or false, especially with respect to predicting these classifications as a function of conditional probability and statistical relevance. This is a highly relevant research question since the influence of probabilistic factors on causal claims and conditionals has been studied extensively, but in separate literatures. We therefore conducted a study where participants classified a given causal claim and the corresponding conditional as true or false, and estimated in addition two probabilistic variables: the conditional probability of the consequent, given the antecedent, and the probability of the consequent simpliciter. Our specific interest was in finding whether these probabilistic features could reliably predict the classification of causal claims/conditionals as true or false.

Our informal discussion at the beginning of this paper has suggested that the truth conditions of indicative conditionals are more demanding than the truth conditions of corresponding tendency causal claims. This claim, expressed in hypotheses H1.a and H1.b, has been supported by our experimental results. This does not exclude that non-causal conditionals such as “if there is smoke, there is fire” can be true even when corresponding causal claims “smoke causes fire” are false.^10^

In line with theoretical expectations and previous research, conditional probability emerges as a reliable predictor for the classification of the conditional. This finding supports a weak, qualitative version of Adams’s Thesis according to which the conditional probability is correlated with high acceptability/classification as true (H2.a). The effect size in the GLMM is very remarkable ($R^2 \in [0.19;0.62]$). Effect size decreases when restricted to the set of causal claims classified as true (H2.b)—$R^2 \in [0.12;0.16]$ in Experiments 1, 2.C and 2.D, and under the medium effect size threshold for Experiment 2.A and 2.B. However, this is to be expected given that in the set of true causal claims, most conditionals are classified as true: it is thus harder to achieve a high effect size than in the more heterogeneous baseline set. The result should therefore not be taken as an argument against the predictive performance of conditional probability.

The third set of hypotheses in our paper concerns the role of statistical relevance. Here, we built on probabilistic theories of causal strength and on statistical relevance accounts of indicative conditionals in order to formulate four more hypotheses. In Experiment 1, we find that the classification of a causal claim and/or the corresponding conditional as true or false is usually—but not always—predicted by measures of statistical relevance (H3.a and H3.b). This finding has, however, not been replicated in Experiment 2.

Finally, statistical relevance is not any more a relevant predictor if the corresponding causal claim is classified as true (H3.d): the relationship between the various measures and the target variable is still statistically significant, but this is to be expected for such a large data set and the effect size is too small to be of theoretical interest ($R^2 < 0.09$ for all statistical relevance measures).

In line with the partial confirmation of H3.a, this suggests that statistical relevance has little effect on top of causal relevance: it is a decent predictor of causal relevance, but when the latter has been established, statistical relevance does not lead to better predictions of the classification of the conditional and is, in any case, inferior to conditional probability as such a predictor.

The following overall picture of the classification of causal conditionals emerges. They are judged as true or highly acceptable only if (not: if and only if) the corresponding tendency causal claim is accepted. However, the best *probabilistic* predictor of their classification is the conditional probability *p*(*E*|*C*), and not a statistical relevance measure.

These results support, all in all, the “orthodox” line of psychological research that emphasizes the importance of conditional probability for predicting how people evaluate and reason with conditionals (e.g., Evans and Over 2004; Over et al. 2007; Over and Cruz 2023). Our results agree with Evidential Support Theory in so far as EST emphasizes the *relevance* of the antecedent for the consequent (see also Skovgaard-Olsen et al. 2016b). However, we do not observe support for a probabilistic operationalization of EST: statistical relevance can act as a proxy for classifying causal claims (full and partial confirmation of H3.a in the two experiments), but not for the classification of the conditional. Statistical relevance predicts the classification of conditionals neither overall nor in the category of tendency causal claims evaluated as true (see the failure of H3.b/c/d).

In other words, we are skeptical that the truth or acceptability of a (causal) conditional can be reliably predicted by purely statistical factors such as the probability *p*(*E*|*C*) and the degree of statistical relevance of *C* for *E*. Something more substantive, which goes beyond statistical association, seems to be required, too. Whether our results support an inferential approach to the semantics of conditionals in general depends on the specific version and the chosen auxiliary assumptions (compare, for example Krzyżanowska et al. 2014; Douven 2015; Douven et al. 2021).

One of the limitations of our study is the exclusion of counterfactual conditionals, whose causal character has been studied extensively in the literature (Lewis 1973a, b; Pearl 2000; Schulz 2017). We conjecture that analogical relations might hold between counterfactual conditionals and *actual* causal claims. Consider, for example, the sentences “Ben’s attending the party caused him to fail the exam” and “If Ben had not gone to the party, he would have passed the exam”. The relationship between such pairs of sentences strikes us as a valuable object for further research (see also Schulz 2011). All in all, the interface of conditionals, causality and probability emerges as an important and fruitful area for future research which eventually may lead to a unified theory for both types of expressions.

## Data Availability

All data and the details of the design are available here: https://osf.io/e25j3/.

## Appendix A. Instructions for the Experiment 1

“**Thank you for participating in our study.** As a Mechanical Turk worker, you should at least have an approval rating of 95% if you want to be reimbursed for your participation. Please read the questions carefully and answer each with a probability score (between **0%** and **100%**) or truth value (’**True**’ or ’**False**’) If you have any questions or comments, please leave them at the end of the survey. You start the survey by pressing the double arrow at the bottom-right corner.”

## Appendix B. List of Scenarios and Questions for the Experiment 1

- John is a middle-aged man, how likely is it that he will be healthy?
- John is a middle-aged man, how likely is it that he will be healthy if he exercises daily?
- Daily exercising causes John to be healthy.
- If John exercises daily, then he will be healthy.
- How likely is it that a random person is skinny?
- How likely is it that a random person is skinny if his/her daily food intake is 4 apples and 3 cucumber sandwiches?
- Eating only 4 apples and 3 cucumber sandwiches a day causes people to become skinny.
- If people only eat 4 apples and 3 cucumber sandwiches a day, then they will become skinny.
- How likely is it that a random person will catch the flu?
- How likely is it that a random person will catch the flu if two-thirds of his/her co-workers already have it?
- Having people around oneself with the flu causes one to catch the flu.
- If people around oneself have the flu, then one will catch the flu.
- How likely is it that a random person has more than 10 friends?
- How likely is it that a random person has more than 10 friends if he/she uses MDMA at parties?
- Using MDMA at parties causes people to have more than 10 friends.
- If people use MDMA at parties, then they will have more than 10 friends.
- How likely is it that the crime rate will decline in the next 5 years?
- How likely is it that the crime rate will decline in the next 5 years if drugs (xtc, cocaine, weed) are legalized?
- Legalizing drugs (xtc, cocaine, and weed) causes the crime rate to decline over the next 5 years.
- If drugs (xtc, cocaine, and weed) are legalized, then the crime rate will decline over the next 5 years.
- How likely is it that the crime rate will decline in the next 5 years?
- How likely is it that the crime rate will decline in the next 5 years if alcohol consumption is made illegal?
- Making alcohol consumption illegal causes the crime rate to decline over the next 5 years.
- If alcohol consumption is made illegal, then the crime rate will decline over the next 5 years.
- How likely is it that the national birth rate (babies born per capita per year) will decline in the next 10 years?
- How likely is it that the national birth rate (babies born per capita per year) will decline in the next 10 years if contraception is mandatory until the age of 21?
- Making contraception mandatory until age 21 causes the national birth rate to decline.
- If contraception is made mandatory, then the national birth rate will decline.
- How likely is it that the national birth rate (babies born per capita per year) will decline in the next 10 years?
- How likely is it that the national birth rate (babies born per capita per year) will decline in the next 10 years if all men start wearing white socks in sandals?
- All men wearing white socks in sandals causes the national birth rate to decline.
- If all men start wearing white socks in sandals, then the national birth rate will decline.
- How likely is it that the national birth rate (babies born per capita per year) will decline in the next 10 years?
- How likely is it that the national birth rate (babies born per capita per year) will decline in the next 10 years if education is mandatory until age 21?
- Making education mandatory until age 21 causes the national birth rate to decline.
- If education is mandatory until age 21, then the national birth rate will decline.
- How likely is it that a random non-fiction book will be an international best seller (3000 copies sold in the first week)?
- How likely is it that a random non-fiction novel will be a best seller (3000 copies sold in the first week) if the author is Stephen Hawking?
- Having Stephen Hawking as the author causes a book to be a best seller.
- If Stephen Hawking is the author, then a book will be a best seller.
- How likely is it that a random non-fiction book will be an international best seller (3000 copies sold in the first week)?
- How likely is it that a random non-fiction novel will be a best seller (3000 copies sold in the first week) if the author is Sarah Palin?
- Having Sarah Palin as the author causes a book to be a best seller.
- If Sarah Palin is the author, then a book will be a best seller.
- John is a man in the late sixties, he eats fast food every day and does not exercise. How likely it is that he will develop a cancer?
- John is a man in the late sixties, he eats fast food every day and does not exercise. How likely it is that he will develop a cancer if he smokes a pack of cigarettes a day since he was a teenager?
- John’s smoking will cause him to develop a cancer.
- If John smokes, he will develop a cancer.
- How likely it is that a random person will develop a cancer?
- How likely it is that a random person, exposed to a high dose of gamma radiation, will develop a cancer?
- Exposure to a high dose of gamma radiation causes cancer.
- If one is exposed to a high dose of gamma radiation, he will develop a cancer.
- How likely it is that a random child will be tall?
- How likely it is that a random child will be tall, given that she or he is drinking a lot of milk every day?
- Drinking a lot of milk everyday causes one to be tall.
- If a child drinks a lot of milk every day, he or she will be tall.
- How likely it is that a random child will be tall?
- How likely it is that a random child will be tall, if she or he is exercising every day?
- Exercising causes one to be tall.
- If one exercises every day, he or she will be tall.
- How likely it is that a random person will become a diabetic?
- How likely it is that a random person who eats three apples a day will become a diabetic?
- Eating three apples a day cause diabetics.
- If one eats three apples a day, he will become a diabetic.
- How likely it is that a random person will have all natural teeth at the age of sixty?
- How likely it is that a random person will have all natural teeth at the age of sixty, if he or she brushes one’s teeth after every meal?
- Brushing one’s teeth after every meal will cause one to have all natural teeth at the age of sixty.
- If one brushes one’s teeth after every meal, she or he will have all natural teeth at the age of sixty.
- How likely it is that a random person will have all natural teeth at the age of sixty?
- How likely it is that a random person will have all natural teeth at the age of sixty, if he or she brushes one’s teeth after every meal and visits a dentist once a month?
- Brushing one’s teeth after every meal and visiting a dentist once a month will cause one to have all natural teeth at the age of sixty.
- If one brushes one’s teeth after every meal and visits a dentist once a month, she or he will have all natural teeth at the age of sixty.
- What is the probability that the approval of the government will decrease in the next few months?
- What is the probability that the approval of the government will decrease in the next few months if the majority party proposes legislation that bans alcohol?
- A legislation proposal which bans alcohol, made by the majority party, will cause a decrease in approval of the government in the next few months.
- If the majority party proposes legislation that bans alcohol, the approval of the government will decrease in the next few months.

## Appendix C. Instructions for the Experiment 2

“**Instructions** You will be asked to evaluate 18 scenarios, each containing 2 questions. Please read the questions carefully and answer each with a probability score (between 0% and 100%) At the end there will be a few socio-demographic questions. The experiment will take about 15 minutes and is concluded with a debriefing on what the experiment was about You start the survey by pressing the double arrow at the bottom-right corner.”

## Appendix D. List of Scenarios and Questions for the Experiment 2

- Suppose that John is a middle-aged man. How likely is it that John is healthy?
- Suppose that John is a middle-aged man who exercises frequently. How likely is it that John is healthy?
- How likely is it that a randomly selected person is skinny?
- Suppose that a randomly selected person eats few fats and carbs. How likely is it that she is skinny?
- How likely is it that a randomly selected person will catch the flu?
- Suppose that many co-workers of a randomly selected person have the flu. How likely is it that she will catch the flu?
- How likely is it that a randomly selected person has more than 10 friends?
- Suppose that a randomly selected person occasionally uses MDMA (xtc) at parties. How likely is it that she has more than 10 friends?
- How likely is it that the crime rate will decline in the next 5 years?
- Suppose that drugs (xtc, cocaine, weed) will be legalized. How likely is it then that the crime rate will decline in the next 5 years?
- How likely is it that the crime rate will decline in the next 5 years?
- Suppose that alcohol consumption will be prohibited. How likely is it then that the crime rate will decline in the next 5 years?
- How likely is it that the national birth rate will decline?
- Suppose that contraception is made mandatory until the age of 21. How likely is it then that the national birth rate will decline?
- How likely is it that the national birth rate will decline?
- Suppose that many men start wearing white socks in sandals. How likely is it then that the national birth rate will decline?
- How likely is it that the national birth rate will decline?
- Suppose that education is made mandatory until age 21. How likely is it then that the national birth rate will decline?
- How likely is it that a physics book will be a bestseller?
- Suppose that Stephen Hawking is the author of a physics book. How likely is it that the book will be a bestseller?
- How likely is it that a physics book will be a bestseller?
- Suppose that Donald Trump is the author of a physics book. How likely is it that the book will be a bestseller?
- Suppose that Bill is a man in his late sixties, he eats fast food every day and does not exercise. How likely is it that he will develop cancer?
- Suppose that Bill is a man in his late sixties. He eats fast food every day, does not exercise, and smokes frequently. How likely is it that he will develop cancer?
- How likely is it that a randomly selected person will develop cancer?
- Suppose that a randomly selected person is exposed to a high dose of gamma radiation. How likely is it that she will develop cancer?
- How likely is it that a randomly selected child will be tall as an adult?
- Suppose that a randomly selected child frequently drinks milk. How likely is it that the child will be tall as an adult?
- How likely is it that a randomly selected child will be tall as an adult?
- Suppose that a randomly selected child exercises frequently. How likely is it that the child will be tall as an adult?
- How likely is it that a randomly selected person will have all natural teeth at the age of sixty?
- Suppose that a randomly selected person regularly brushes her teeth. How likely is it that she will have all natural teeth at the age of sixty?
- How likely is it that a randomly selected person will have all natural teeth at the age of sixty?
- Suppose that a randomly selected person frequently visits a dentist. How likely is it that she will have all natural teeth at the age of sixty?
- How likely is it that the government’s approval rate will decrease?
- Suppose that the government proposes a law that bans alcohol. How likely is it then that the government’s approval rate will decrease?

## Footnotes

1 Following Hitchcock (2001), we will define tendency causal claims as causal assertions that do not imply the truth of its arguments. Tendency causal claims are distinguished from *actual* causal claims, such as “James Dean’s recklessness caused his accident.” These are typically not expressed through indicative conditionals but through counterfactual structures, for instance, “If James Dean had not been reckless, he would not have died” (Lewis 1973a, b; Pearl 2000). As discussed in Hitchcock (2001), the tendency vs. actual distinction is orthogonal to the type vs. token distinction (see Sikorski 2022 for discussion). All the causal claims used in our experiments will be tendency causal claims, encompassing both type and token claims.

2 This does not include conditionals which connect two effects of a common cause, such as “if I have fever now, my muscles will ache tomorrow”.

3 Inferential conditionals can be further divided into inductive, abductive, and deductive conditionals (see, e.g., Douven and Verbrugge 2010). Inductive conditionals express inductive inferences, deductive conditionals express deductive inferences, and abductive conditionals express abductive inferences. “If this paper was rejected, then it must have been bad” is an example of an abductive conditional.

4 Note that these are cut-offs, not intended to be evaluated by a statistical tests. Meaning, that H1.b, for instance, will be rejected if the acceptance has any value below 90%.

5 Crupi and Iacona also make a case distinction between positive and negative relevance, but we simplify their measure for purposes of exposition.

6 As pointed out by an anonymous referee, the classification of the conditional is not always straightforward. An example used by the reviewer is, “If I have fever now, I’ll develop muscle aches tomorrow.” It is an inferential conditional whose grammatical form is identical to a causal conditional. A corresponding causal claim is false; therefore, if we included such conditionals, H1.a would come out false. We therefore excluded these conditionals from our experimental vignettes, at the price of making the support in favor of H1.a less surprising. As noted by the editor, Paul Égré, an alternative strategy would be to use reductive theories of causality (e.g., Eells 1991 or Pearl 2000) to define causal conditionals in non-causal terms, such as dependence or temporal order.

7 This may be read as ambiguous with respect to the probability of the conditional, as opposed to the conditional probability of the consequent. However, while these two quantities can differ in principle (e.g., Douven 2016; Skovgaard-Olsen et al. 2016b), they will typically be aligned when the antecedent is *relevant* for the consequent, e.g., part of the same discourse. This is the case for a majority of our vignettes. Moreover, questions about the probability of the conditional are typically asked in the form “Please rate the probability of the following sentence: ...”. We slightly altered the phrasing in Experiment 2, but for the above reasons, we consider the risk that the phrasing affected the results rather low.

8 Please note that the statistical relevance measures do not have the same scale and range. The coefficients can be meaningfully interpreted as the increase of the dependent value with each 1.0 increase of the statistical relevance measure. Look at the *z*-scores of the measures to compare them on the adequacy of predicting the dependent variable.

9 Because for Experiment 2.C and 2.D the outcome variable is continuous, the model is actually a General Linear Mixed Model

10 See e.g., Johnson-Laird and Byrne (2002) for an experiment which uses such conditionals.
